# Integration analysis for novel lncRNA markers predicting tumor recurrence in human colon adenocarcinoma

**DOI:** 10.1186/s12967-019-2049-2

**Published:** 2019-08-30

**Authors:** Fangyao Chen, Zhe Li, Changyu Deng, Hong Yan

**Affiliations:** 1 Department of Epidemiology and Biostatistics, School of Public Health, Xi’an Jiaotong University Health Science Center, 76 Yanta Xilu Road, Xi’an, 710061 Shaanxi China; 2 First Affiliated Hospital of Xi’an Jiaotong University, 277 Yanta Xilu Road, Xi’an, 710061 Shaanxi China; 3 Department of Preventive Medicine, Shantou University Medical College, 22 Xinling Road, Jinping District, Shantou, 515041 Guangdong China

**Keywords:** lncRNA, Colon adenocarcinoma, Tumor recurrence, Integrative analysis, ceRNA network

## Abstract

**Background:**

Numerous evidence has suggested that long non-coding RNA (lncRNA) acts an important role in tumor biology. This study focuses on the identification of novel prognostic lncRNA biomarkers predicting tumor recurrence in human colon adenocarcinoma.

**Methods:**

We obtained the research data from The Cancer Genome Atlas (TCGA) database. The interaction among different expressed lncRNA, miRNA and mRNA markers between colon adenocarcinoma patients with and without tumor recurrence were verified with miRcode, starBase and miRTarBase databases. We established the lncRNA–miRNA–mRNA competing endogenous RNA (ceRNA) network based on the verified association between the selected markers. We performed the functional enrichment analysis to obtain better understanding of the selected lncRNAs. Then we use multivariate logistic regression to identify the prognostic lncRNA markers with covariates. We also generated a nomogram predicting tumor recurrence risk based on the identified lncRNA biomarkers and clinical covariates.

**Results:**

We included 12,727 lncRNA, 1881 miRNA and 47,761 mRNA profiling and clinical features for 113 colon adenocarcinoma patients obtained from the TCGA database. After filtration, we used 37 specific lncRNAs, 60 miRNAs and 148 mRNAs in the ceRNA network analysis. We identified five lncRNAs as prognostic lncRNA markers predicting tumor recurrence in colon adenocarcinoma, in which four of them were identified for the first time. Finally, we generated a nomogram illustrating the association between the identified lncRNAs and the tumor recurrence risk in colon adenocarcinoma.

**Conclusions:**

The four newly identified lncRNA biomarkers might be potential prognostic biomarkers predicting tumor recurrence in colon adenocarcinoma. We recommend that further clinical and fundamental researches be conducted on the identified lncRNA markers.

## Background

Colorectal carcinoma (CRC) is among the most common cancers with high morbidity and mortality among all malignancies and the most common CRC is colon adenocarcinoma (CA) [1]. It was reported that over 70% of CA patients would develop tumor recurrence within 24 months after surgery [2] and tumor recurrence still acts as one of the most severe risk factors to overall survival of CA patients [3]. Thus, the issue of tumor recurrence following a primary CA becomes very important [4]. It is essential to identify prognostic markers in order to study the biological mechanism in CA and identify the candidate targets for therapy.

Long non-coding RNAs (lncRNAs), with lengths of at least 200 nucleotides, modulate gene expression at the post-transcriptional level [5]. With the innovations in RNA sequencing technologies and computational biology, recent findings suggest that lncRNAs are involved in the biological process of cancer development [6]. Numerous studies on the role of lncRNAs in various types of cancers have been performed, and several lncRNA biomarkers have been identified to be related to the development, diagnosis and overall survival of various cancers [7–11]. Recently, lncRNA *HOTAIR* has been identified to be related to the overall survival in CA [12]. LncRNA *ATB* is associated with poor prognosis of CRC [13]. LncRNA *CCAT1* is reported to be of clinical value in the diagnosis of CA [14]. All these studies suggest a potential value of lncRNAs in the prognosis and diagnosis of CA.

The competing endogenous RNA (ceRNA) hypothesis was presented as a new model demonstrating the association between non-coding and coding RNAs and accepted as one of the most efficient tools in lncRNAs research [10]. It has been widely utilized in the identification of diagnostic and prognostic lncRNA markers in various cancers [15–18].

Few studies have focused on the ceRNA network related to tumor recurrence in CA [19–22]. Thus, in this study, we aim to establish the lncRNA–miRNA–mRNA ceRNA network for the tumor recurrence of CA to identify novel prognostic lncRNA biomarkers for the prediction of tumor recurrence in CA and to achieve better understanding of the role of lncRNAs in CA based on the RNA sequencing data obtained from The Cancer Genome Atlas (TCGA) database.

## Materials

### Data profile

We obtained the RNA sequencing (including miRNA, lncRNA and mRNA) measurements and clinical characteristics of CA patients from The Cancer Genome Atlas (TCGA) database (https://cancergenome.nih.gov/), an open source of information to identify novel biomarkers in cancer research, using R package “*TCGAbiolinks*” in June 2018. As a result, 12,727 lncRNA, 1881 miRNA and 47,761 mRNA profiling were obtained.

We excluded patients with missing information in tumor recurrence status, tumor location, venous status, lymphatic invasion status, histology type, pathology stage, TNM stage or age at diagnosis. We also excluded records without lncRNA, miRNA or mRNA measurements. Finally, 113 records were included in this study. The maximum follow-up time was 10.37 years (3780 days) with medium follow-up time equal to 1.34 years (488 days). During the follow-up, 6 out of 113 individuals were recorded as deceased.

### Data pre-processing

Since the obtained lncRNA and miRNA expression measurements were not normally distributed, we performed a log transformation to normalize and correct their positively skewed distributions. Suppose *x*_*ij*_ is the expression for *jth* lncRNA or miRNA expression of *ith* individual, the transformed value of *j*th lncRNA or miRNA expression of *i*th individual would equal to ln(*x*_*ij*_) (if *x*_*ij*_ > 0) or 0 (if *x*_*ij*_ = 0). The transformation can be expressed with:

$$  {\text{Transformed}}\_{\text{expression}} = {\text{ }}\left\{ {\begin{array}{*{20}c}    {\ln \left( {x_{{ij}} } \right),} & {if\quad x_{{ij}}  > 0}  \\    {0,} & {if\quad x_{{ij}}  = 0}  \\   \end{array} } \right. $$

Then, before incorporating into multi-variate analysis, to obtain a better explanation to the coefficient obtained in regression analysis, we transform the log-transformed lncRNA expressions into binary variables according to whether the log-transformed expression was higher (up-regulated) or lower (down-regulated) than its log-transformed mean.

The mRNA profiling obtained from the TCGA database were normally distributed, therefore, no pre-processing was performed on the mRNA expressions.

### Statistical analysis

All analyses were performed through R (version 3.4.4, the R Foundation for Statistical Computing, Vienna, Austria). Clinical and demographic characteristics were tested (with *α *= 0.05) by Chi-square test (gender, pathology stage and tumor site), Mann–Whitney test (TNM stage) and *t*-test (age at diagnosis). We also used *t*-test to select lncRNAs, miRNAs (log-transformed) and mRNAs with different expression levels between CA patients with and without tumor recurrence (with *α *= 0.01), the *p*-value obtained were adjusted with the BH method [23]. Prognostic lncRNA markers were identified based on the adjusted ORs obtained from multi-variate logistic regression.

### Establishment of lncRNA–miRNA–mRNA ceRNA network

We constructed the lncRNA–miRNA–mRNA ceRNA network to identify miRNAs associated mRNAs based on the interaction among lncRNA, miRNA and mRNA that were verified based on the miRcode (http://www.mircode.org/) [24], starBase (http://starbase.sysu.edu.cn/) [25], and miRTarBase (http://mirtarbase.mbc.nctu.edu.tw/php/index.php) databases [26]. First, we use *t*-test (with BH correction) to select lncRNAs, miRNAs and mRNAs with different expression levels between cases with and without tumor recurrence. Then, the differentially expressed lncRNAs, miRNAs and mRNAs, which have been verified in the miRcode, starBase and miRTanBase databases, were incorporated into the construction of lncRNA–miRNA–mRNA ceRNA network. The lncRNA–miRNA–mRNA ceRNA network was conducted using the Cytoscape software (version 3.6.1, National Institute of General Medical Science, Bethesda, MD, US) [27]. We also used the “*clusterMaker2*” [28] application within in the Cytoscape software to identify subnetworks through the Markov Cluster Algorithm (MCL clustering) [28].

### Functional annotation analysis

We performed the functional annotation analysis using “*clueGO*” [29] application within the Cytoscape software. Gene Ontology (GO) analysis was performed based on the GO database (http://www.geneontology.org/) [30]. Pathway enrichment analysis was performed based on the Kyoto Encyclopedia of Genes and Genomes database (KEGG) (https://www.genome.jp/kegg/pathway.html) [31] and the Reactome (https://www.reactome.org/) databases [32].

### Nomogram construction

Finally, we generated a nomogram predicting tumor recurrence risk for asymptotic CA patient based on the lncRNA markers and clinical features identified in the current study using R package “*rms*”. The prediction performance was evaluated by the ROC analysis and C-index.

## Results

### Characteristics of included patients

Out of the 113 patients, 50 (44.2%) were males. 67 (59.2%) patients were diagnosed with stage I/II CA, 46 (40.8%) were at stage III/IV, 98 (30.9%). 29 (25.7%) patients developed venous invasion, 56 (49.6%) developed lymphatic invasion. The average age at diagnosis for patients with and without tumor recurrence were 71.95 ± 11.93 and 65.33 ± 12.26 years old. The primary diagnosed sites and detailed TNM stage information of included CA patients are shown in Table 1. Out of the 113 CA patients included in this study, 98 (86.7%) developed tumor recurrence.

**Table 1:** Clinical characteristics of included CA patients

Factor	Categories	Tumor status		Total	*p*-value
		Free	Recurred		
Gender	Female	10	53	63	0.361^a^
	Male	5	45	50	
Venous invasion	No	11	73	84	0.924^a^
	Yes	4	25	29	
Lymphatic invasion	No	9	48	57	0.427^a^
	Yes	6	50	56	
Pathology stage	I/II	10	57	67	0.053^a^
	III/IV	5	41	46	
T stage	T1	0	3	3	0.151^b^
	T2	2	19	21	
	T3	9	64	73	
	T4	4	12	16	
	N0	10	58	68	0.295^b^
N stage	N1	4	19	23	
	N2	1	21	22	
M stage	M0	14	81	95	0.293^a^
	M1	1	17	18	
Tumor site	Ascending colon	4	20	24	0.009^a^
	Cecum	7	18	25	
	Sigmoid colon	1	42	43	
	Transverse colon	3	7	10	
	Other^c^	0	11	11	
Age at diagnosis	Mean	65.33	71.95	–	0.049^d^
	SD	12.26	11.93	–	

^a^With Chi-square test

^b^With Mann–Whitney test

^c^Including descending colon, hepatic flexure and splenic flexure

^d^With t-test

### Selection of different expressed RNA sequencing measurements

We selected the lncRNA, miRNA and mRNA measurements with different expression levels between patients with and without tumor recurrence through independent *t*-tests with *p*-value adjusted through the BH method [19]. As a result, we selected 61 lncRNA, 167 miRNA and 354 mRNA measurements with adjusted *p*-value less than 0.01. The selected lncRNAs, miRNAs and mRNAs are contained in Additional file 1: S1 (sheets 1–3).

### Establishment of lncRNA–miRNA–mRNA ceRNA network

**Fig. 1:**
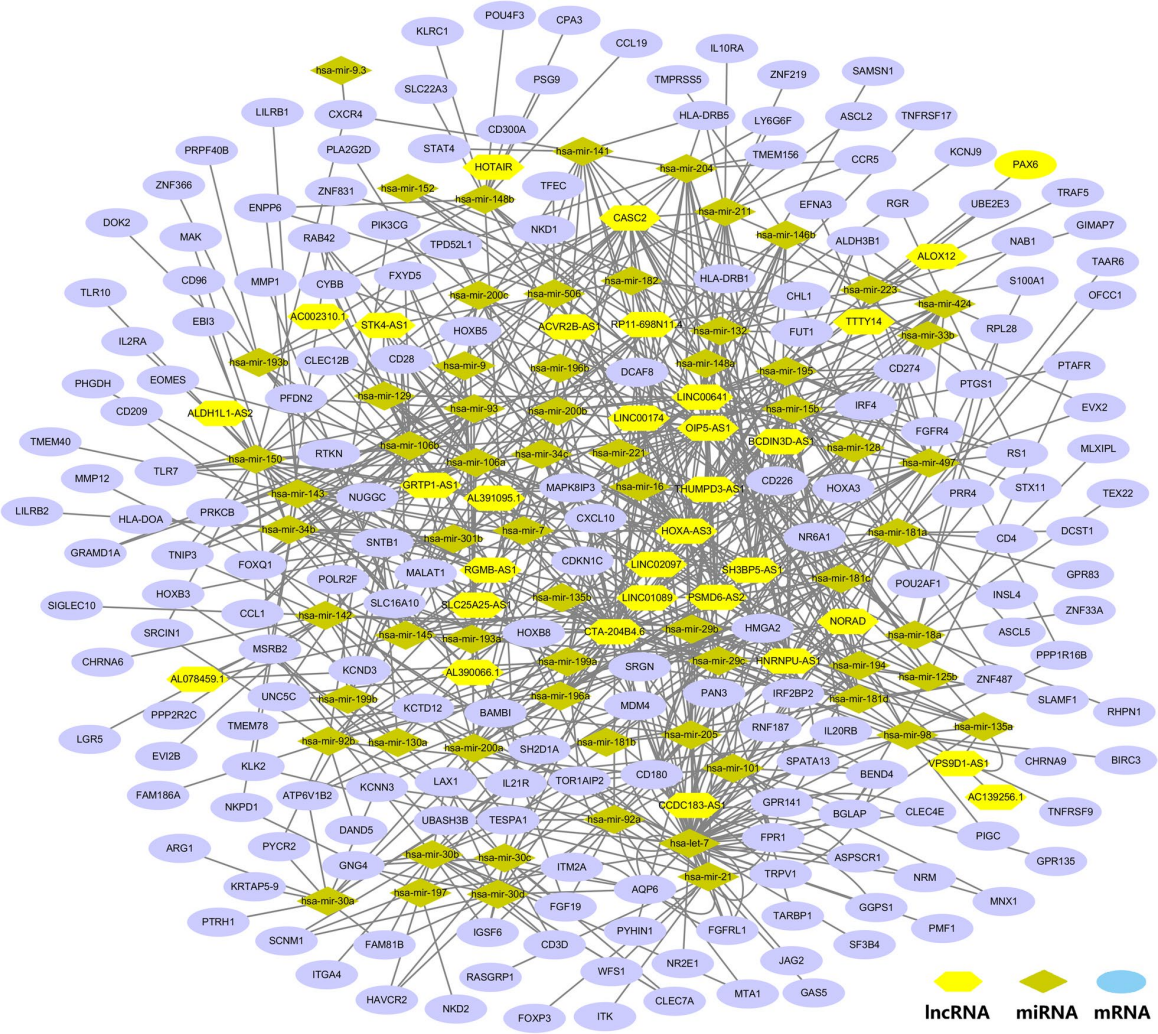
The lncRNA-miRNA-mRNA ceRNA network. The hexagon represents lncRNAs, the circle stands for the mRNAs and the diamond is for the miRNAs

We used the miRcode, starBase and miRTarBase databases to verify the interaction relationship between the different expressed lncRNA, miRNA and mRNA markers. Based on the verified interaction relationship, we conducted the lncRNA–miRNA–mRNA ceRNA network analysis to reveal the association between selected lncRNAs and miRNAs as shown in Fig. 1. The detailed information is shown in Tables 2 and 3. As a result, the ceRNA network indicated that 60 particular miRNAs interacted with 33 specific lncRNAs and 148 mRNAs.

**Table 2:** lncRNA targeted miRNA verified in CA

lncRNA	Targeted miRNA
*AC002310.1*	*hsa*-*mir*-*129*
*AC139256.1*	*hsa*-*mir*-*194*
*ACVR2B*-*AS1*	*hsa*-*mir*-*125b, hsa*-*mir*-*141, hsa*-*mir*-*143, hsa*-*mir*-*182, hsa*-*mir*-*200a, hsa*-*mir*-*7*
*AL078459.1*	*hsa*-*mir*-*145, hsa*-*mir*-*199a, hsa*-*mir*-*7*
*AL390066.1*	*hsa*-*mir*-*106a, hsa*-*mir*-*141, hsa*-*mir*-*150, hsa*-*mir*-*200a, hsa*-*mir*-*205, hsa*-*mir*-*9, hsa*-*mir*-*93, hsa*-*mir*-*145, hsa*-*mir*-*29b, hsa*-*mir*-*29c*
*AL391095.1*	*hsa*-*mir*-*106a, hsa*-*mir*-*141, hsa*-*mir*-*150, hsa*-*mir*-*200a, hsa*-*mir*-*205, hsa*-*mir*-*9, hsa*-*mir*-*93, hsa*-*mir*-*145, hsa*-*mir*-*29b, hsa*-*mir*-*29c*
*ALDH1L1*-*AS2*	*hsa*-*mir*-*145*
*BCDIN3D*-*AS1*	*hsa*-*mir*-*125b, hsa*-*mir*-*146b, hsa*-*mir*-*182, hsa*-*mir*-*18a, hsa*-*mir*-*205*
*CASC2*	*hsa*-*mir*-*101, hsa*-*mir*-*106a, hsa*-*mir*-*125b, hsa*-*mir*-*141, hsa*-*mir*-*143, hsa*-*mir*-*15b, hsa*-*mir*-*16, hsa*-*mir*-*181a, hsa*-*mir*-*181c, hsa*-*mir*-*181d, hsa*-*mir*-*193a, hsa*-*mir*-*193b, hsa*-*mir*-*194, hsa*-*mir*-*195, hsa*-*mir*-*200a, hsa*-*mir*-*200b, hsa*-*mir*-*200c, hsa*-*mir*-*204, hsa*-*mir*-*205, hsa*-*mir*-*21, hsa*-*mir*-*211, hsa*-*mir*-*33b, hsa*-*mir*-*34b, hsa*-*mir*-*34c, hsa*-*mir*-*424, hsa*-*mir*-*497, hsa*-*mir*-*506, hsa*-*mir*-*93*
*CCDC183*-*AS1*	*hsa*-*let*-*7, hsa*-*mir*-*205, hsa*-*mir*-*205, hsa*-*mir*-*29b, hsa*-*mir*-*29c, hsa*-*mir*-*98*
*CTA*-*204B4.6*	*hsa*-*let*-*7, hsa*-*mir*-*106a, hsa*-*mir*-*106b, hsa*-*mir*-*128, hsa*-*mir*-*129, hsa*-*mir*-*130a, hsa*-*mir*-*132, hsa*-*mir*-*135a, hsa*-*mir*-*135b, hsa*-*mir*-*142, hsa*-*mir*-*143, hsa*-*mir*-*145, hsa*-*mir*-*146b, hsa*-*mir*-*150, hsa*-*mir*-*15b, hsa*-*mir*-*16, hsa*-*mir*-*181a, hsa*-*mir*-*181c, hsa*-*mir*-*181d, hsa*-*mir*-*195, hsa*-*mir*-*196a, hsa*-*mir*-*196b, hsa*-*mir*-*197, hsa*-*mir*-*200b, hsa*-*mir*-*200c, hsa*-*mir*-*204, hsa*-*mir*-*205, hsa*-*mir*-*211, hsa*-*mir*-*221, hsa*-*mir*-*29b, hsa*-*mir*-*29c, hsa*-*mir*-*301b, hsa*-*mir*-*30a, hsa*-*mir*-*30b, hsa*-*mir*-*30c, hsa*-*mir*-*30d, hsa*-*mir*-*33b, hsa*-*mir*-*34b, hsa*-*mir*-*34c, hsa*-*mir*-*424, hsa*-*mir*-*497, hsa*-*mir*-*92a, hsa*-*mir*-*92b, hsa*-*mir*-*93, hsa*-*mir*-*98*
*GRTP1*-*AS1*	*hsa*-*mir*-*106a, hsa*-*mir*-*106b, hsa*-*mir*-*146b, hsa*-*mir*-*150, hsa*-*mir*-*93*
*HNRNPU*-*AS1*	*hsa*-*mir*-*125b, hsa*-*mir*-*129, hsa*-*mir*-*132, hsa*-*mir*-*135a, hsa*-*mir*-*135b, hsa*-*mir*-*145, hsa*-*mir*-*145, hsa*-*mir*-*15b, hsa*-*mir*-*16, hsa*-*mir*-*181a, hsa*-*mir*-*181c, hsa*-*mir*-*181d, hsa*-*mir*-*18a, hsa*-*mir*-*195, hsa*-*mir*-*199a, hsa*-*mir*-*204, hsa*-*mir*-*205, hsa*-*mir*-*211, hsa*-*mir*-*424, hsa*-*mir*-*497, hsa*-*mir*-*506*
*HOXA*-*AS3*	*hsa*-*mir*-*106a, hsa*-*mir*-*106a, hsa*-*mir*-*106b, hsa*-*mir*-*125b, hsa*-*mir*-*132, hsa*-*mir*-*141, hsa*-*mir*-*143, hsa*-*mir*-*150, hsa*-*mir*-*15b, hsa*-*mir*-*16, hsa*-*mir*-*18a, hsa*-*mir*-*195, hsa*-*mir*-*200a, hsa*-*mir*-*204, hsa*-*mir*-*205, hsa*-*mir*-*211, hsa*-*mir*-*29b, hsa*-*mir*-*29c, hsa*-*mir*-*34b, hsa*-*mir*-*34c, hsa*-*mir*-*424, hsa*-*mir*-*497, hsa*-*mir*-*506, hsa*-*mir*-*7, hsa*-*mir*-*9, hsa*-*mir*-*93*
*HOTAIR*	*hsa*-*mir*-*148b*
*LINC00174*	*hsa*-*mir*-*130a, hsa*-*mir*-*15b, hsa*-*mir*-*16, hsa*-*mir*-*195, hsa*-*mir*-*199a, hsa*-*mir*-*204, hsa*-*mir*-*211, hsa*-*mir*-*301b, hsa*-*mir*-*424, hsa*-*mir*-*497*
*LINC00641*	*hsa*-*mir*-*129, hsa*-*mir*-*132, hsa*-*mir*-*135a, hsa*-*mir*-*135b, hsa*-*mir*-*145, hsa*-*mir*-*146b, hsa*-*mir*-*148a, hsa*-*mir*-*148b, hsa*-*mir*-*152, hsa*-*mir*-*15b, hsa*-*mir*-*16, hsa*-*mir*-*195, hsa*-*mir*-*200b, hsa*-*mir*-*200c, hsa*-*mir*-*204, hsa*-*mir*-*211, hsa*-*mir*-*221, hsa*-*mir*-*424, hsa*-*mir*-*497, hsa*-*mir*-*506, hsa*-*mir*-*7*
*LINC01089*	*hsa*-*let*-*7, hsa*-*mir*-*128, hsa*-*mir*-*129, hsa*-*mir*-*135a, hsa*-*mir*-*135b, hsa*-*mir*-*145, hsa*-*mir*-*15b, hsa*-*mir*-*16, hsa*-*mir*-*193a, hsa*-*mir*-*193b, hsa*-*mir*-*195, hsa*-*mir*-*34b, hsa*-*mir*-*34c, hsa*-*mir*-*424, hsa*-*mir*-*497, hsa*-*mir*-*98*
*LINC02097*	*hsa*-*mir*-*125b, hsa*-*mir*-*128, hsa*-*mir*-*143, hsa*-*mir*-*33b*
*NORAD*	*hsa*-*let*-*7, hsa*-*mir*-*101, hsa*-*mir*-*181a, hsa*-*mir*-*181c, hsa*-*mir*-*181d, hsa*-*mir*-*182, hsa*-*mir*-*194, hsa*-*mir*-*199a, hsa*-*mir*-*204, hsa*-*mir*-*205, hsa*-*mir*-*211, hsa*-*mir*-*92a, hsa*-*mir*-*92b, hsa*-*mir*-*98*
*OIP5*-*AS1*	*hsa*-*let*-*7, hsa*-*mir*-*130a, hsa*-*mir*-*132, hsa*-*mir*-*141, hsa*-*mir*-*143, hsa*-*mir*-*145, hsa*-*mir*-*146b, hsa*-*mir*-*148a, hsa*-*mir*-*148b, hsa*-*mir*-*150, hsa*-*mir*-*152, hsa*-*mir*-*15b, hsa*-*mir*-*16, hsa*-*mir*-*181a, hsa*-*mir*-*181c, hsa*-*mir*-*181d, hsa*-*mir*-*18a, hsa*-*mir*-*194, hsa*-*mir*-*195, hsa*-*mir*-*196a, hsa*-*mir*-*196b, hsa*-*mir*-*200a, hsa*-*mir*-*200b, hsa*-*mir*-*200c, hsa*-*mir*-*205, hsa*-*mir*-*221, hsa*-*mir*-*223, hsa*-*mir*-*301b, hsa*-*mir*-*424, hsa*-*mir*-*497, hsa*-*mir*-*7, hsa*-*mir*-*98*
*PSMD6*-*AS2*	*hsa*-*mir*-*106a, hsa*-*mir*-*135a, hsa*-*mir*-*135b, hsa*-*mir*-*141, hsa*-*mir*-*148a, hsa*-*mir*-*148b, hsa*-*mir*-*181a, hsa*-*mir*-*181c, hsa*-*mir*-*181d, hsa*-*mir*-*182, hsa*-*mir*-*200a, hsa*-*mir*-*204, hsa*-*mir*-*211, hsa*-*mir*-*9, hsa*-*mir*-*93*
*RGMB*-*AS1*	*hsa*-*mir*-*129, hsa*-*mir*-*143, hsa*-*mir*-*150, hsa*-*mir*-*34c, hsa*-*mir*-*7, hsa*-*mir*-*92a, hsa*-*mir*-*92b*
*RP11*-*698N11.4*	*hsa*-*mir*-*106a, hsa*-*mir*-*128, hsa*-*mir*-*132, hsa*-*mir*-*142, hsa*-*mir*-*148a, hsa*-*mir*-*148b, hsa*-*mir*-*152, hsa*-*mir*-*223, hsa*-*mir*-*34b, hsa*-*mir*-*34c, hsa*-*mir*-*7*
*SH3BP5*-*AS1*	*hsa*-*mir*-*101, hsa*-*mir*-*106a, hsa*-*mir*-*125b, hsa*-*mir*-*132, hsa*-*mir*-*143, hsa*-*mir*-*146b, hsa*-*mir*-*15b, hsa*-*mir*-*16, hsa*-*mir*-*181a, hsa*-*mir*-*181c, hsa*-*mir*-*181d, hsa*-*mir*-*182, hsa*-*mir*-*195, hsa*-*mir*-*199a, hsa*-*mir*-*200a, hsa*-*mir*-*204, hsa*-*mir*-*211, hsa*-*mir*-*223, hsa*-*mir*-*34b, hsa*-*mir*-*34c, hsa*-*mir*-*424, hsa*-*mir*-*497, hsa*-*mir*-*93*
*SLC25A25*-*AS1*	*hsa*-*mir*-*150, hsa*-*mir*-*193a, hsa*-*mir*-*193b, hsa*-*mir*-*199a, hsa*-*mir*-*205, hsa*-*mir*-*34b, hsa*-*mir*-*34c, hsa*-*mir*-*9*
*STK4*-*AS1*	*hsa*-*mir*-*106a, hsa*-*mir*-*106a, hsa*-*mir*-*106b, hsa*-*mir*-*182, hsa*-*mir*-*93*
*THUMPD3*-*AS1*	*hsa*-*mir*-*143, hsa*-*mir*-*145, hsa*-*mir*-*146b, hsa*-*mir*-*15b, hsa*-*mir*-*16, hsa*-*mir*-*181a, hsa*-*mir*-*181c, hsa*-*mir*-*181d, hsa*-*mir*-*195, hsa*-*mir*-*221, hsa*-*mir*-*29b, hsa*-*mir*-*29c, hsa*-*mir*-*424, hsa*-*mir*-*497, hsa*-*mir*-*9*
*TTTY14*	*hsa*-*mir*-*33b*
*VPS9D1*-*AS1*	*hsa*-*mir*-*135a, hsa*-*mir*-*135a, hsa*-*mir*-*135b*

**Table 3:** miRNA targeted mRNA verified in miRTarBase databases

miRNA	Targeted mRNA
*hsa*-*let*-*7*	*AQP6, ASPSCR1, BEND4, BGLAP, CLEC4E, DCAF8, FGFRL1, FPR1, GGPS1, GPR141, HMGA2, IRF2BP2, JAG2, KCTD12, MDM4, NR2E1, NR6A1, NRM, PMF1, PPP1R16B, RS1, SF3B4, SPATA13, TOR1AIP2, TOR1AIP2, TRPV1, UBASH3B*
*hsa*-*mir*-*101*	*BEND4, CD180, CD180, GPR135, IL20RB, MNX1, TOR1AIP2*
*hsa*-*mir*-*106a*	*CD28, CLEC12B, DCAF8, FOXQ1, FXYD5, HMGA2, KCND3, NUGGC, PRKCB, RAB42, RAB42, TLR7, TNIP3*
*hsa*-*mir*-*106b*	*BAMBI, CCL1, CD28, CLEC12B, CYBB, DCAF8, EOMES, FOXQ1, FXYD5, GRAMD1A, KCND3, NUGGC, PRKCB, RAB42, TLR7, TNIP3*
*hsa*-*mir*-*125b*	*BGLAP, PTAFR*
*hsa*-*mir*-*128*	*ASCL5, CYBB, IL21R*
*hsa*-*mir*-*129*	*HOXB8*
*hsa*-*mir*-*130a*	*ATP6V1B2, FOXQ1, HOXB3, SNTB1*
*hsa*-*mir*-*132*	*CD226, CHL1, FUT1, KCNJ9, S100A1, SAMSN1, TNFRSF17*
*hsa*-*mir*-*135a*	*LAX1, BGLAP*
*hsa*-*mir*-*141*	*HLA*-*DRB1, HLA*-*DRB5, HOXB5, MALAT1, MDM4, STAT4*
*hsa*-*mir*-*142*	*CHRNA6, EVI2B, HMGA2, KCND3, KCTD12, LGR5, PIK3CG, PPP2R2C, PRKCB, SH2D1A, SIGLEC10, TNIP3, TOR1AIP2*
*hsa*-*mir*-*143*	*BAMBI, ENPP6, IL2RA, KLK2, LILRB1, MAPK8IP3, NKPD1, PHGDH, SLC16A10, TMEM40*
*hsa*-*mir*-*145*	*CD28, HMGA2, MMP1, MMP12, RTKN, SLC16A10, SNTB1*
*hsa*-*mir*-*146b*	*MALAT1, PTGS1, TMPRSS5*
*hsa*-*mir*-*148a*	*HLA*-*DRB1, PAN3*
*hsa*-*mir*-*148b*	*CCL19, CD300A, CPA3, CYBB, ENPP6, IL21R, KLRC1, PIK3CG, POU4F3, PSG9, SLC22A3*
*hsa*-*mir*-*150*	*CD96, CXCR4, FXYD5, MSRB2, NKD1, NUGGC, SRCIN1, TLR10, TLR7, TNIP3*
*hsa*-*mir*-*152*	*CD274*
*hsa*-*mir*-*15b*	*ALDH3B1, BAMBI, CD180, CD274, FGFR4, HOXA3, IL20RB, IRF4, NAB1, NR6A1, POU2AF1, RS1, STX11*
*hsa*-*mir*-*16*	*CD226, HMGA2, INSL4*
*hsa*-*mir*-*181a*	*CD4, CHL1, DCST1, GPR83, HMGA2, NR6A1, OFCC1, PRR4, RNF187, S100A1, SRGN, TAAR6, ZNF487*
*hsa*-*mir*-*181b*	*HMGA2, NR6A1, RNF187, SH2D1A, SRGN, ZNF487*
*hsa*-*mir*-*181c*	*HMGA2, KCNN3, NR6A1, RNF187, SRGN, ZNF487*
*hsa*-*mir*-*181d*	*HMGA2, NR6A1, RNF187, SRGN, ZNF487*
*hsa*-*mir*-*182*	*CHL1, ZNF831*
*hsa*-*mir*-*18a*	*DCAF8, MLXIPL, PTGS1, RHPN1, RNF187, RPL28, TEX22, ZNF33A*
*hsa*-*mir*-*193a*	*FGF19, RTKN*
*hsa*-*mir*-*193b*	*DOK2, FXYD5, PFDN2, PRPF40B, RTKN, ZNF366*
*hsa*-*mir*-*194*	*AQP6, CD274, HMGA2, MAPK8IP3, SLAMF1, SPATA13*
*hsa*-*mir*-*195*	*ALDH3B1, ALOX12, CD180, CD274, FGFR4, HOXA3, IRF4, NKD1, NR6A1, POU2AF1, RS1, UBE2E3*
*hsa*-*mir*-*196a*	*ATP6V1B2, CXCL10, FUT1, HMGA2, HOXB8, NR6A1, TMEM78, TRPV1*
*hsa*-*mir*-*196b*	*HMGA2, HOXB8, NR6A1, PIK3CG*
*hsa*-*mir*-*197*	*FPR1, NKD2, NKPD1*
*hsa*-*mir*-*199a*	*CDKN1C, LAX1, POLR2F, SLC16A10, SNTB1, TOR1AIP2, UNC5C*
*hsa*-*mir*-*199b*	*LAX1, POLR2F, SLC16A10, SNTB1, TOR1AIP2, UNC5C*
*hsa*-*mir*-*200a*	*HOXB5, MALAT1, MDM4, MNX1, UBASH3B*
*hsa*-*mir*-*200b*	*CD274, HOXB5, MALAT1, MDM4, TPD52L1*
*hsa*-*mir*-*200c*	*CXCL10, HOXB5, MALAT1, POLR2F, TPD52L1*
*hsa*-*mir*-*204*	*CCR5, CD28, CXCR4, CYBB, HLA*-*DRB1, HLA*-*DRB5, HMGA2, IRF2BP2, LY6G6F, MALAT1, TMEM156*
*hsa*-*mir*-*205*	*BAMBI, GPR141, IGSF6, ITM2A, PYHIN1*
*hsa*-*mir*-*21*	*CCL1, CXCL10, DCAF8, FGFRL1, FOXP3, GAS5, ITK, KLK2, MDM4, PAN3, RASGRP1, SLC16A10, TOR1AIP2, WFS1*
*hsa*-*mir*-*211*	*ASCL2, CCR5, CD28, HLA*-*DRB1, HLA*-*DRB5, IL10RA, IRF2BP2, LY6G6F, RPL28, TMEM156, ZNF219*
*hsa*-*mir*-*221*	*CDKN1C, HOXB5, PTAFR*
*hsa*-*mir*-*223*	*CD226, GIMAP7, PAX6, PPP1R16B, TRAF5, UBE2E3*
*hsa*-*mir*-*29b*	*IRF2BP2, KCTD12*
*hsa*-*mir*-*29c*	*CD274, IRF2BP2, TARBP1, TESPA1*
*hsa*-*mir*-*301b*	*ATP6V1B2, CLEC12B, FOXQ1, HLA*-*DOA, HOXB3, LILRB2, SNTB1*
*hsa*-*mir*-*30a*	*ARG1, ATP6V1B2, BAMBI, FAM81B, HMGA2, IL21R, KCNN3, KRTAP5*-*9, MDM4, PTRH1, PYCR2*
*hsa*-*mir*-*30b*	*AQP6, CD180, CD3D, CLEC7A, FAM81B, FGF19, GNG4, HAVCR2, IGSF6, IL21R, ITGA4, LAX1, MSRB2, MTA1, SCNM1, TOR1AIP2*
*hsa*-*mir*-*30c*	*AQP6, CD180, CD3D, CLEC7A, FGF19, GNG4, HAVCR2, IGSF6, LAX1, MSRB2, SCNM1, TOR1AIP2*
*hsa*-*mir*-*30d*	*AQP6, AQP6, CD3D, CLEC7A, FAM81B, GNG4, HAVCR2, IGSF6, IL21R, LAX1, MSRB2, RNF187, SCNM1, TOR1AIP2*
*hsa*-*mir*-*33b*	*EVX2, HMGA2*
*hsa*-*mir*-*34b*	*CD209, CD274, EBI3, MAK, POLR2F, TESPA1, ZNF831*
*hsa*-*mir*-*34c*	*MDM4, PLA2G2D*
*hsa*-*mir*-*424*	*ALDH3B1, CD180, CD274, FGFR4, HOXA3, NR6A1, RGR, RS1*
*hsa*-*mir*-*497*	*ALDH3B1, CD180, CD274, EFNA3, EVX2, FGFR4, HOXA3, IRF4, NR6A1, RS1*
*hsa*-*mir*-*506*	*ENPP6, FOXQ1, HMGA2, TFEC*
*hsa*-*mir*-*7*	*MDM4, PFDN2, SPATA13*
*hsa*-*mir*-*9.3*	*CXCR4*
*hsa*-*mir*-*92a*	*FGF19, KCNN3, MTA1, TESPA1*
*hsa*-*mir*-*92b*	*CD180, CD226, CDKN1C, DAND5, FAM186A, HMGA2, LAX1, NUGGC, PYCR2, SRCIN1*
*hsa*-*mir*-*93*	*CCL1, CD28, CLEC12B, DCAF8, FOXQ1, FXYD5, GRAMD1A, KCND3, NUGGC, PFDN2, PRKCB, RAB42, TLR7, TNIP3, UNC5C*
*hsa*-*mir*-*98*	*BIRC3, CHRNA9, HMGA2, PIGC, TNFRSF9*

### Functional enrichment analysis

**Fig. 2:**
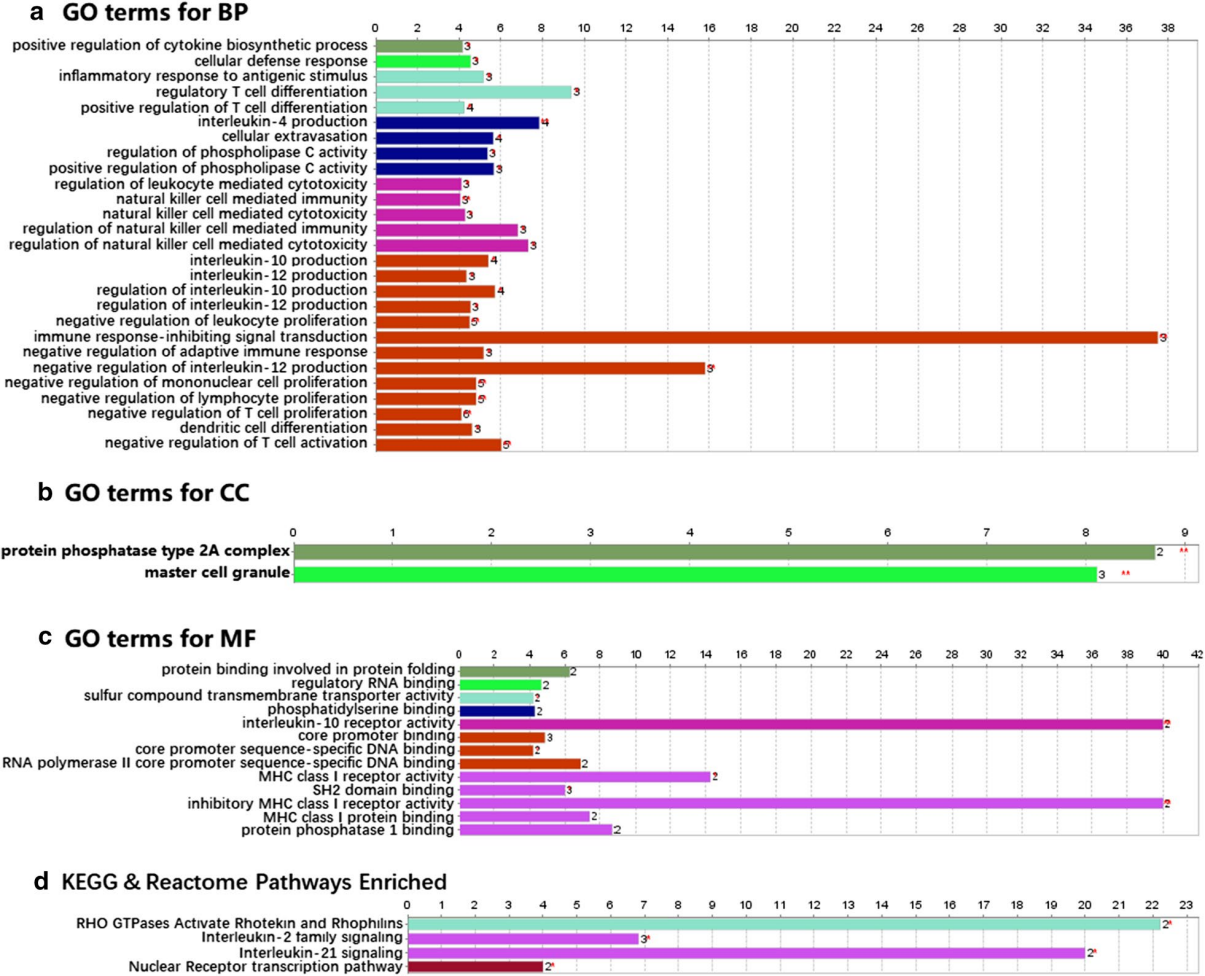
Barplots generated in enrichment analyses. **a** barplot of gene ontology enriched in biological process (BP); **b** barplot of gene ontology enriched in cellular component (CC); **c** barplot of gene ontology enriched in molecular function (MF); **d** barplot of KEGG and Reactome pathway analysis; The length of bar reflected the percent of the gene cluster

In order to obtain a deep understanding on the selected genes, we performed the functional enrichment analysis to the intersection mRNAs as shown in Fig. 2. It revealed that the enriched GO terms for biological process (BP) were mainly related to the several immune processes including the regulation of the differentiation and proliferation for several immune cells (T cell, mononuclear cell and leukocyte), the regulation of cytotoxicity and immunity mediated by natural killer cell and leukocyte, the differentiation of dendritic cell, the regulation of interleukin-10 and -12 and the regulation of cytokine, as shown in Fig. 2a. The associated cell component (CC) included protein phosphatase type 2A complex and mast cell granule as shown in Fig. 2b. The enriched GO terms for molecular function (MF) were mainly about core promoter binding, interleukin-10 activity, MHC class 1 activity, as shown in Fig. 2c.

The pathways enriched based on the KEGG and Reactome databases are shown in Fig. 2d. The revealed pathways included RHO GTPases Activate Rhotekin and Rhophilins pathway, Interleukin-21 signaling pathway, Interleukin-2 family signaling pathway and Nuclear Receptor transcription pathway.

### Subnetwork analysis

**Fig. 3:**
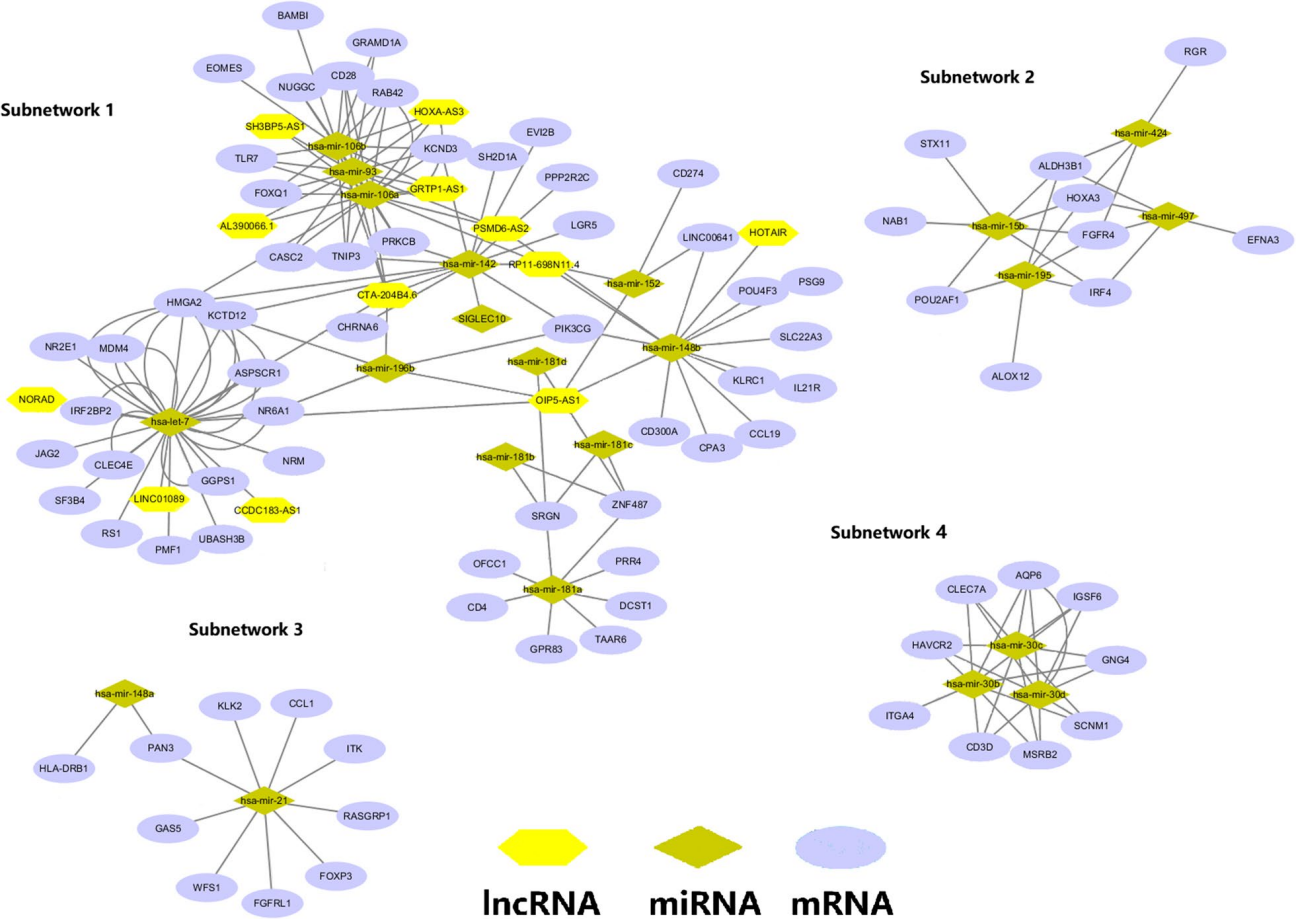
Subnetwork analysis based on the main ceRNA network. A total of 37 subnetworks were identified while only subnetworks with at least 10 nodes (subnetwork 1 to 4) were selected. The hexagon represents lncRNAs, the circle stands for the mRNAs and the diamond is for the miRNAs

**Fig. 4:**
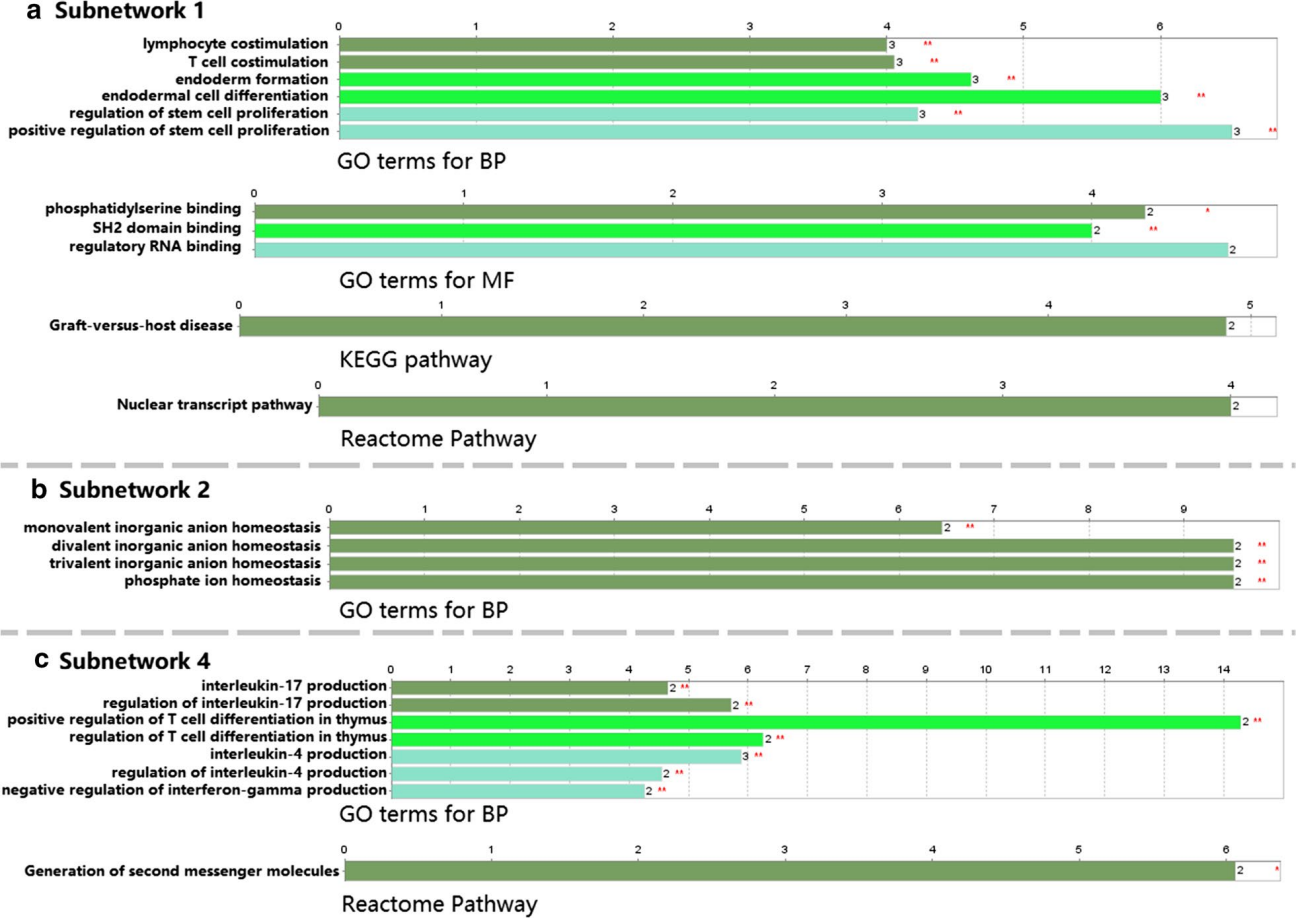
Functional enrichments for the genes in the subnetworks. **a** Enriched GO terms for biological process (BP), molecular function (MF) and pathways for subnetwork 1; **b** enriched GO terms for biological process (BP) for subnetwork 2; **c** enriched GO terms for biological process (BP), and pathway for subnetwork 4

We used the “*clusterMaker2*” [28] application within the Cytoscape software to identify the subnetworks from the main ceRNA network through the MCL clustering approach [28]. As a result, we identified 37 distinct subnetworks. Based on the number of nodes in each subnetworks, after filtration, we selected 4 subnetworks with at least 10 nodes, as shown in Fig. 3. We also performed the functional enrichment analysis for the genes in the selected subnetworks; the results are shown in Fig. 4. The enriched GO terms for BP in subnetwork 1 and 4 were mainly about several human immune processes, which consistent with that obtained from the main network.

### Identification of prognostic lncRNA markers

We applied the univariate logistic regression model to estimate the ORs for patients with different expression types of the each lncRNA markers in developing tumor recurrence or not separately. Among the 39 lncRNAs identified in ceRNA network, 8 lncRNAs yielded statistical significance (*p *< 0.05) in univariate logistic regression and were incorporated into multivariate logistic regression with clinical covariates.

Finally, 5 lncRNAs (*CASC2*, *AL078459.1*, *AL390066.1*, *STK4*-*AS1* and *HOXA*-*AS3*) yielded statistical significance (*p *< 0.05) in multivariate logistic analysis with age at diagnosis and tumor pathology stage as covariates. The result suggests that these lncRNA markers might act as prognostic predictors for the tumor recurrence in CA. The adjusted ORs of the 5 identified prognostic lncRNAs, in particular, indicated that the up-regulation of *CASC2* (*OR *= 1.225, 95%CI 1.061–5.025), *AL078459.1* (*OR *= 2.923, 95% CI 1.504–7.946), *AL390066.1*, (*OR *= 2.311, 95% CI 1.182–4.764) *STK4*-*AS1* (*OR *= 3.611, 95% CI 1.328–3.030) and *HOXA*-*AS3* (*OR *= 2.511, 95% CI 1.026–4.415) may be associated with the development of tumor recurrence of CA.

### Nomogram for tumor recurrence prediction

**Fig. 5:**
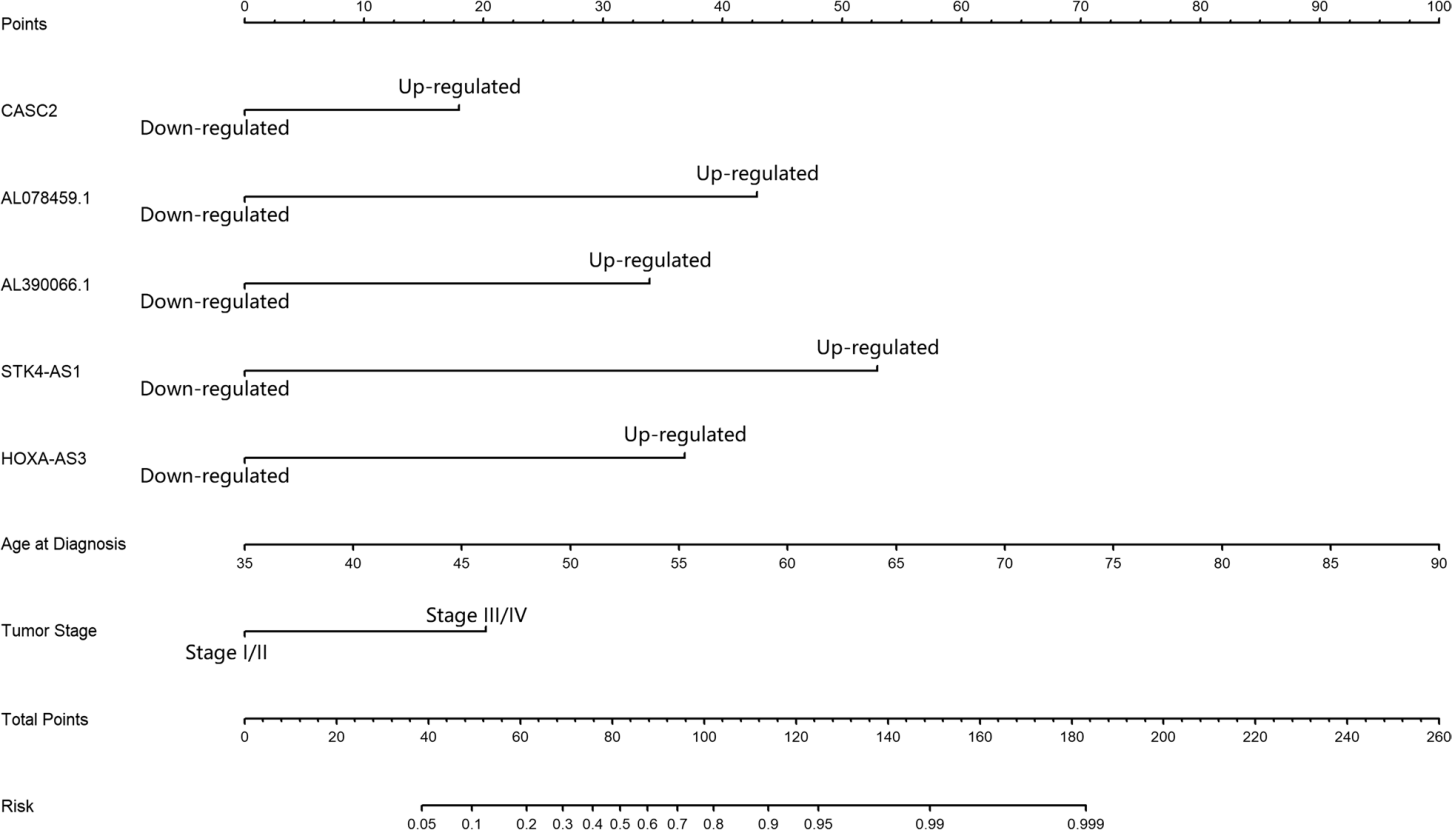
Nomogram of tumor recurrence risk (probability) prediction for asymptomatic CA patients. To estimate the rate for a real patient, identify the patient’s regulation type for each lncRNA markers and clinical characteristic status, draw a line from the observed status for each factors straight upwards to the Points axis to obtain the points a factor. Repeat this procedure until scores for all factors were decided. Sum the points corresponding to lncRNAs, and clinical characters and locate the summed point on the Total Points axis. Draw a line straight down to the Risk axis to check the rate for the particular patient

Finally, we generated a simple-to-use nomogram based on the 5 prognostic lncRNA markers and clinical characteristics (pathology stage and age at diagnosis) of CA patients as shown in Fig. 5. It could provide useful information in prediction of tumor recurrence for asymptomatic CA patients based on multivariate logistic regression. The C-index for the model was 0.895 and the area under the ROC for the model is 0.885 (95% CI based on bootstrap method: 0.836–0.935). Both C-index and the ROC analysis suggested a good predict performance.

## Discussion

### General comments

Differential expression of lncRNAs has been widely identified in various cancers. Published studies have revealed that lncRNAs have key roles in vital biological functions of cancers. However, only few studies have described the role of lncRNA profiles in tumor recurrence of CA [19–22]. In this study, we focused on the identification of novel prognostic markers for the tumor recurrence of CA based on the RNA sequencing data from the TCGA database. We have constructed the lncRNA–miRNA–mRNA ceRNA network to clarify the unknown ceRNA regulatory network in tumor recurrence of CA. As a result, 5 lncRNAs (*CASC2*, *AL078459.1*, *AL390066.1*, *STK4*-*AS1* and *HOXA*-*AS3*) were identified through the ceRNA network and multivariate logistic regression. We also performed functional enrichment analysis to investigate the molecular role of the identified lncRNA biomarkers.

For the 5 identified lncRNA markers, *CASC2* has been reported to be associated with colorectal cancer [33–35]. Studies have revealed that the deregulation of *CASC2* by miRNA *hsa*-*mir*-*21* and *hsa*-*mir*-*18a* increases the proliferation and migration of cancer cells in colorectal cancer [33–35]. The link between the *CASC2* and the prognosis of CA suggested in published literatures were consistent with the results in this study. This also indicated that the results of our research were reasonable. For *STK4*-*AS1*, it was associated with protein coding gene *STK4* while the down-regulation of *STK4* was associated with the invasion and migration of colorectal cancer [36]. This also suggests that the association between *STK4*-*AS1* and the prognosis of CA revealed in this study was reasonable. For *HOXA*-*AS3*, though it has not been identified in CA before, the up-regulation of *HOXA*-*AS3* was reported to be associated with tumor progression and poor prognosis in glioma [37]. In this study, we found *STK4*-*AS1* and *HOXA*-*AS3* were also related to tumor recurrence in CA. For lncRNAs *AL078459.1* and *AL390066.1*, though no functional roles have been reported in CA before this work, in our study, have been identified to be related to the tumor recurrence in CA.

To obtain a deep understanding of the selected lncRNA markers, the functional enrichment analyses were performed. The enriched in GO terms were related with several human immune process. IL-10 and IL-12, as representative immune factors, play an important role in inflammation and tumorigenesis [38], and published research also suggested a potential relationship between them and the progression of CRC [39]. Tumor infiltrating T-cells was related to the microsatellite instability and the prognosis of CRC [40]. Natural killer cell plays an important role in the anti-cancer defense, and has great potential in cancer immunotherapy in cancer immunotherapy [41]. Dendritic cell [42], tumor infiltrating mononuclear cell [43] and tumor infiltrating mast cell [44] were all associated with the progression and prognosis of CRC. MHC class I is a major component of tumor-associated antigen presenting system, which responded to a large number of chemotherapeutic agents in the treatment of CRC [45]. For the enriched pathways, RHO GTPases Activate Rhotekin and Rhophilins pathway was associated with the development and progression of several solid malignancies including CRC [46, 47], Interleukin-21 signaling pathway was associated with the development of colitis-associated CRC [48] and Interleukin-2 family signaling pathway acts important role in current anti-tumor immunotherapy [49]. The immune infiltration is closely associated with prognosis of CRC [50, 51], the results of enrichment analysis suggested that the genes in the ceRNA network were associated with several important human immune processes, thus may be associated with the clinical outcome of CRC.

For the subnetwork analysis, we identified 37 distinct subnetworks while only those with at least 10 nodes were selected (as shown in Fig. 3). Then, we performed the functional enrichment analysis for the genes involved in each subnetworks. For subnetwork 1, the enriched GO terms for BP were mainly about the co-stimulation of T cell and lymphocyte cell, the differentiation of endodermal cell and the proliferation of stem cell. For subnetwork 3, the enriched biological processes were mainly about the regulation of IL-17 and IL4, the differentiation of thymus and the regulation of the production of interferon gamma, all related to the progression and prognosis of CA [52–55]. Most of biological processes enriched for the subnetworks were about human immune process and this was consistent with the biological processes enriched for the main network. For the enriched terms for MF in subnetwork 1, though different from that enriched from the main network, phosphatidylcholine has been found to involve in the growth of CRC cell [56]. The enriched pathways based on the KEGG and Reactome databases for subnetwork 2 (nuclear transcript pathway) were consistent with those enriched in the main network. The consistency in functional enrichments between the subnetworks and the main network suggested good robustness of our analysis.

The nomogram generated in this study was simple-to-use and would be useful in estimating the tumor recurrence risk for asymptotic patients with CA. It also visualized the associations between each prognostic lncRNA and clinical features (stage and age at diagnosis) and the tumor prognosis of CA patients.

Recently, many studies on the identification of prognostic genes in CA based on the TCGA database have been reported and their findings are then verified with functional experiments in succession [57–60]. Those studies all suggested a good reliability of the TCGA database in identifying new prognostic gene signatures for the prognosis in cancer studies and the potential value of the results obtained in the current work.

### Limitations

Firstly, our study was a preliminary work. In this study, we obtained the RNA sequencing and clinical data from public database, but no clinical samples were involved. Thus, the importance of the selected markers still need to be validated in future cohort study. Then, though we tried to incorporated as many clinical factors as possible in our analysis, some important factors were still not available, for example, treatment information (such as chemotherapy, surgery and radiotherapy), and living habits (such as smoking or drinking habits). These might cause potential bias in analysis.

## Conclusion

In this study, we identified five prognostic lncRNA markers for the prediction of tumor recurrence in CA based on ceRNA hypothesis and data obtained from the TCGA database, in which, four of the selected lncRNA markers (*AL078459.1*, *AL390066.1*, *STK4*-*AS1* and *HOXA*-*AS3*) were identified for the first time. The hub genes in the network were annotated with functional gens sets associated with colorectal cancer and the mechanism of tumor progression and invasion. Our work also provides a simple-to-use nomogram predicting the tumor recurrence risk for asymptotic CA patients based on the lncRNA markers identified in this study with clinical covariates. Though clinical validation is still needed, it is reasonable to conclude that these miRNAs are worthwhile for further study as novel candidate prognostic biomarkers for the survival of CA.

## Supplementary information

**Additional file 1.** Differently expressed genes (lncRNAs, miRNAs and mRNAs) selected between CA patients with and without tumor recurrence.

## Data Availability

The datasets analyzed during the current study are available at the TCGA database (https://cancergenome.nih.gov/).

## Abbreviations

TCGA: The Cancer Genome Atlas
CA: colon adenocarcinoma
CRC: colorectal carcinoma
lncRNA: long non-coding RNA
ceRNA: competing endogenous RNA
KEGG: Kyoto Encyclopedia of Genes and Genomes
GO: Gene Ontology
BP: biological process
MF: molecular function
CC: cellular component
